# Recurrent Exacerbations and Evolution into Polymyositis in a Patient with Interstitial Pneumonia with Autoimmune Features: A Case Report and Literature Review

**DOI:** 10.3390/medicina59020330

**Published:** 2023-02-10

**Authors:** Chien-Tzu Huang, Tsan-Teng Ou, Jui-Sheng Hsu, Chih-Hung Cheng, Chau-Chyun Sheu

**Affiliations:** 1 Division of Hematology and Oncology, Department of Internal Medicine, Kaohsiung Medical University Hospital, Kaohsiung 807, Taiwan; 2 Division of Rheumatology, Department of Internal Medicine, Kaohsiung Medical University Hospital, Kaohsiung 807, Taiwan; 3 Department of Medical Imaging, Kaohsiung Medical University Hospital, Kaohsiung 807, Taiwan; 4 Department of Radiology, School of Medicine, College of Medicine, Kaohsiung Medical University, Kaohsiung 807, Taiwan; 5 Division of Pulmonary and Critical Care Medicine, Department of Internal Medicine, Kaohsiung Medical University Hospital, Kaohsiung 807, Taiwan; 6 Department of Internal Medicine, School of Medicine, College of Medicine, Kaohsiung Medical University, Kaohsiung 807, Taiwan

**Keywords:** anti-Jo-1, glucocorticoid pulse therapy, interstitial pneumonia with autoimmune features, polymyositis

## Abstract

Interstitial pneumonia with autoimmune features (IPAF) is a new disease entity proposed in 2015. Numerous questions regarding IPAF require clarification, including diagnostic criteria, standard managements for stable disease and exacerbation, and prognosis. We report a case of a 67-year-old Asian woman who presented with progressive dyspnea. Chest computed tomography (CT) scans revealed nonspecific interstitial pneumonia. Serologic testing indicated positive anti-Jo-1 without presence of extrathoracic manifestations. An IPAF diagnosis was made after a multidisciplinary discussion. The patient experienced a severe exacerbation requiring mechanical ventilation, and she was successfully salvaged with methylprednisolone pulse therapy and single-dose cyclophosphamide. During the one-year follow-up, she reported bilateral leg muscle weakness with noticeably elevated serum creatine kinase, suggesting polymyositis. The development of malignancy was also noted 15 months after the initial presentation, and the patient eventually died. This report demonstrated successful salvage treatment with glucocorticoid pulse therapy for IPAF with acute exacerbation. However, the maintenance therapy failed to control disease progression. The treatment strategies for exacerbation and stable disease in IPAF remain unknown and need further studies. Given the high risk of evolution into a defined connective tissue disease (CTD), regular evaluation of the clinical features and biomarkers of CTDs is essential for patients with IPAF.

## 1. Introduction

In 2015, the European Respiratory Society (ERS)/American Thoracic Society (ATS) Task Force on Undifferentiated Forms of Connective Tissue Disease (CTD)-associated Interstitial Lung Disease (ILD) proposed the new term “interstitial pneumonia with autoimmune features” (IPAF), which describes patients who have both ILD and autoimmune features but do not meet the current criteria for any defined CTD [1]. The proposal to introduce the term IPAF has attracted considerable interest and promoted discussions between pulmonologists and rheumatologists. With an increasing number of reports and clinical studies being published, our understanding of IPAF is increasing [2]. However, numerous questions in this field require further clarification, including diagnostic criteria, standard management for stable disease and exacerbation, and prognosis of IPAF [3]. We present a challenging case of a patient with severe IPAF who was successfully salvaged through methylprednisolone pulse therapy and single-dose cyclophosphamide for exacerbation. She eventually developed polymyositis after 1-year follow-up. We reviewed the literature and summarized the incidence and types of CTDs evolved from IPAF. This case also highlights the importance of long-term surveillance for CTDs in patients with IPAF.

## 2. Case Report

**Figure 1:**
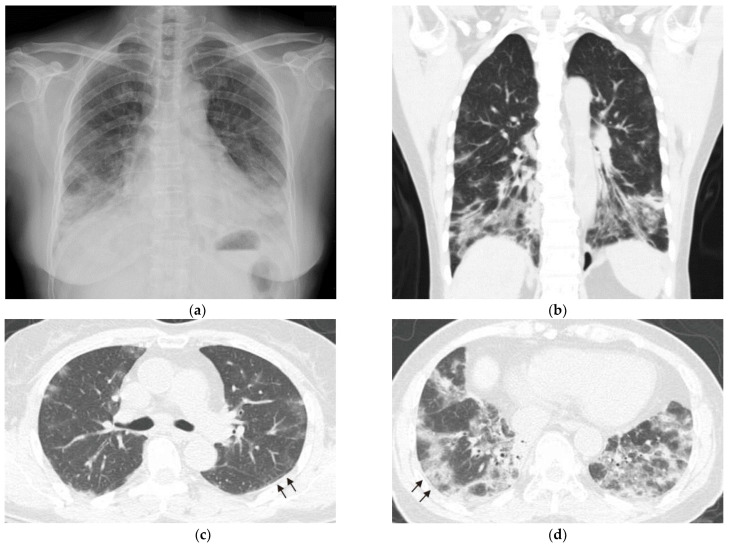
Chest imaging at initial presentation (**a**) Chest radiograph revealed reticular opacities and consolidations in bilateral lower lungs. (**b**–**d**) Computed tomography scans revealed subpleural sparing patterns (arrows) and multifocal ground-glass opacities in bilateral basal distribution, indicating nonspecific interstitial pneumonia.

A 67-year-old non-smoking woman was admitted to our hospital for progressive dyspnea that lasted for 1 week. She had a history of chronic hepatitis C, diabetes mellitus, and endometrial cancer after hysterectomy without recurrence in 2 years. She was well until 2 months before admission, when she experienced fever, dyspnea, and productive cough. She was admitted to another hospital, where she underwent a chest radiograph that revealed reticular opacities and consolidations in the bilateral lower lungs (Figure 1a). Chest computed tomography (CT) revealed a predominance of multifocal ground-glass opacities in bilateral basal lungs with subpleural sparing patterns, suggesting nonspecific interstitial pneumonia (NSIP; Figure 1b–d). Blood testing was positive for anti-CTD antibodies, and detailed serologic testing was not conducted. Her condition improved substantially after treatment with antibiotics and methylprednisolone for possible pneumonia and ILD. After the patient was discharged from hospital, she received prednisolone (10 mg/day) and azathioprine (50 mg/day) at a rheumatology outpatient clinic. No specific diagnosis of CTD was made.

One week before admission, the patient visited our pulmonology outpatient clinic for deteriorating exertional dyspnea. She had received home oxygen therapy since discharge, and her oxygen saturation was 85% on ambient air. Preliminary serologic testing revealed positive anti-Jo-1. The complete blood count and other biomarkers of CTD were normal. The patient had no muscle or skin manifestations suggestive of inflammatory myopathy. She denied myalgia, muscle weakness, arthralgia, skin rash, Raynaud’s phenomenon, mechanic’s hands, or dysphagia. She did not have a family history of autoimmune disorders or personal occupational or environmental exposures. Under the suspicion of IPAF, she was referred to the rheumatologist for a detailed screening for CTDs. Home oxygen therapy and continued treatment with prednisolone and azathioprine were also suggested to the patient.

**Figure 2:**
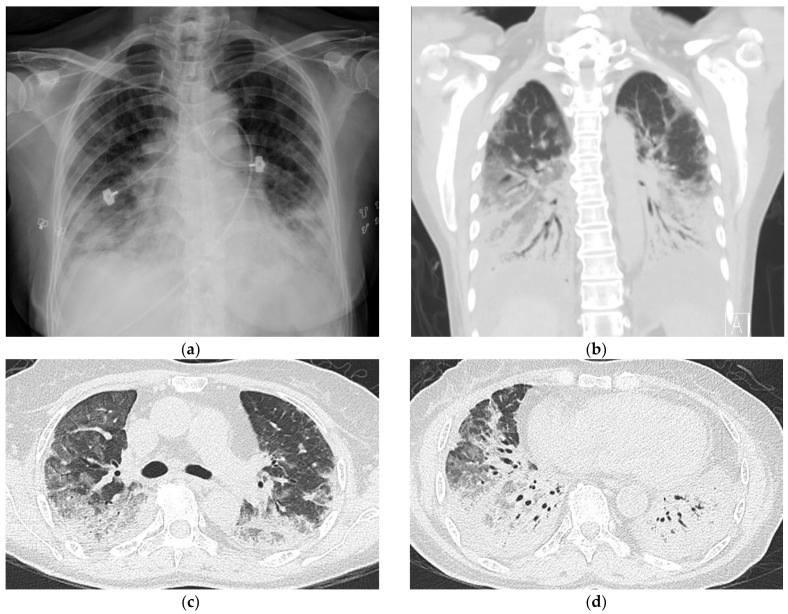
Chest imaging upon admission to the intensive care unit (**a**) Chest radiograph revealed new development of bibasilar consolidations. (**b**–**d**) Computed tomography scans revealed consolidations and increased ground-glass opacities over the bilateral lungs.

Two days before admission, she presented at our emergency department with fever, cough, and progressive dyspnea. An examination revealed that she had tachypnea and an oxygen saturation of 66% while breathing ambient air. She received oxygen therapy through a non-rebreathing mask. Her white-cell count was 12,800 per µL (reference: 4140–10,520 per µL), and her C-reactive protein was 59 mg/L (reference: <5 mg/L). Her hemoglobin, platelet, serum procalcitonin, B-type natriuretic peptide, creatine kinase, liver, and renal functions were all normal. Detailed serologic testing disclosed a positive anti-Jo-1 of 89 EliAU/mL (reference: <7 EliAU/mL) and anti-Ro52 of 3+ (reference: negative). Her rheumatoid arthritis factor, anti-La, anti-SCL-70, anti-ds DNA, anti-MDA-5, and other myositis-specific autoantibodies (MSAs) were all negative. A chest radiograph revealed consolidations in the bilateral lower lungs (Figure 2a).

After the patient was admitted to an intensive care unit, we shifted oxygen supplementation to a high-flow nasal cannula that delivered a fraction of inspired oxygen (FiO_2_) of 80%. Chest CT revealed consolidations and ground-glass opacities over the bilateral lungs with peripheral and lower lung zone predominance (Figure 2b–d). Sputum cultures showed no growth of bacteria, fungus or mycobacteria. Given the features of positive anti-Jo-1 and anti-Ro52, the absence of extrathoracic manifestations, and the failure to meet specific CTD criteria, a diagnosis of IPAF was made after a multidisciplinary discussion involving two pulmonologists, a rheumatologist, and a radiologist. Other diagnoses, including bacterial pneumonia, adult respiratory distress syndrome, cardiogenic pulmonary edema, and cryptogenic organizing pneumonia were further excluded based on her clinical presentations and laboratory data. Additionally, her clinical features were unable to fulfill the diagnosis of anti-synthetase syndrome based on the criteria proposed by Solomon et al. [4]. The patient received methylprednisolone pulse therapy (500 mg/day for 3 days), followed by one dose of 500 mg cyclophosphamide. On the 5th day of hospitalization, she was intubated for severe hypoxemia. She also received treatment with prone positioning and broad-spectrum antibiotic piperacillin/tazobactam (4.5 g every 8 h), and her oxygen saturation improved gradually. On the 16th day of hospitalization, she was extubated successfully and discharged on the 26th day.

A pulmonary function test (PFT) conducted 2 months after hospital discharge revealed a restrictive pattern with a severe reduction in diffusing capacity for carbon monoxide (DLCO; 2.52 L, 38% of predicted), total lung capacity (TLC; 2.32 L, 55% of predicted), and forced vital capacity (FVC; 1.24 L, 55% of predicted). The forced expiratory volume in 1 s (FEV_1_)/FVC was 91.71%. The patient received regular follow-up at rheumatology and pulmonology outpatient clinics and was treated with azathioprine (50 mg/day), hydroxychloroquine (400 mg/day), prednisolone (7.5 mg/day), and as-needed supplemental oxygen at home. A follow-up PFT conducted 4 months later disclosed declining TLC (1.8 L, 51.4% of predicted) and FVC (0.88 L, 39% of predicted) despite maintenance therapy.

Twelve months after initial presentation, the patient reported proximal muscle weakness in the bilateral lower limbs. Her serum creatine kinase was remarkably elevated (1166 IU/L), suggesting polymyositis on the basis of the 2017 European League Against Rheumatism /American College of Rheumatology (EULAR/ACR) classification criteria for idiopathic inflammatory myopathies. The dose of oral prednisolone was increased from 7.5 to 25 mg/day. Shortly thereafter, the patient had another episode of exacerbation, and methylprednisolone pulse therapy (500 mg/day for 2 days) was administered again. On the 8th day of hospitalization, her symptoms ameliorated, and her serum creatine kinase decreased from 1166 to 360 IU/L. Three months later, she experienced a third exacerbation and received another course of methylprednisolone pulse therapy (500 mg/day for 3 days); however, she did not fully recover from her hypoxemic respiratory failure. Subsequently, she was discharged after a prolonged hospitalization and received home oxygen therapy. A follow-up PFT after hospital discharge indicated a progressive decrease in TLC (0.92 L, 21% of predicted) and FVC (0.69 L, 31% of predicted).

Furthermore, a newly developed right upper lung nodule and a left neck lymphadenopathy were detected 15 months after initial presentation. The pathology of lymph node aspiration confirmed the nature of malignancy. However, the patient refused any further diagnostic procedures. Her hypoxemia progressed thereafter, and she died approximately 19 months after initial presentation.

## 3. Discussion

We report a case of IPAF with recurrent exacerbations, which was successfully alleviated through methylprednisolone pulse therapy. An evolution into polymyositis and the development of malignancy were subsequently detected during the clinical course of the patient. The classification criteria of IPAF proposed by the ERS/ATS in 2015 consist of three domains: an extrathoracic clinical domain, serologic domain, and intrathoracic morphologic domain [1]. To date, studies have examined several retrospective cohorts [5,6,7,8,9,10,11,12] to clarify the clinical features of IPAF. These cohorts included a heterogeneous group with a trend of female predominance and participants who were in their mid-50s and mid-60s, mostly non-smoking, and exhibiting NSIP patterns (as revealed through high-resolution CT); these findings are consistent with the characteristics exhibited by our patient. A recent review acknowledged that revision of IPAF criteria is required in the future given increasing published case and cohort reports. This review also provided a summary of the clinical, serological, and morphological domains, as well as treatments and outcomes among numerous IPAF cohorts [13].

Our patient met the criteria of IPAF in serologic and morphologic domains, including positive anti-Jo-1 and a NSIP pattern on CT scans. Alternative diagnoses, such as bacterial pneumonia, acute pulmonary edema, and adult respiratory distress syndrome, were excluded through negative results on sputum culture, normal level of procalcitonin and B-type natriuretic peptide, and a favorable response to treatment with corticosteroid and cyclophosphamide. It is remarkable that we excluded anti-synthetase syndrome in the initial multidisciplinary discussion. The rheumatologist’s original view was mainly based on the Solomon’s criteria [4]. However, there remains no consensus definition for anti-synthetase syndrome. According to the Connor’s criteria [14], the presence of ILD and positive anti-Jo-1 without any other extrapulmonary features in our patient was sufficient to make a diagnosis of anti-synthetase syndrome. Moreover, the differential diagnosis between IPAF and anti-synthetase syndrome remains a considerable challenge due to the overlapping criteria and features. In order to overcome these limitations, EULAR/ACR is currently working on a project with the aim to develop consensus-driven classification criteria for anti-synthetase syndrome [15].

IPAF may represent an early phase of a CTD. The evidence on the incidence of IPAF evolving into a CTD is inconsistent. Ito et al. reported that 12.2% of patients with IPAF experienced evolution into a characterizable CTD during a median follow-up period of 4.5 years [8]. Sambataro et al. conducted a prospective study of 61 patients with IPAF, of which 18.8% developed a specific CTD over the 1-year follow-up [16]. Alevizos et al. also reported that patients with IPAF exhibited a 14 times higher risk of developing a CTD than did patients without IPAF [17]. Seven studies [8,9,16,17,18,19,20] have reported specific CTDs evolved from IPAF, as summarized in Table 1. Collectively, the results of the seven aforementioned studies indicate that idiopathic inflammatory myopathies are the most common diseases, followed by rheumatoid arthritis, systemic sclerosis, and Sjögren syndrome. Our patient was categorized as having initial IPAF that evolved to polymyositis 1 year after initial presentation. Because IPAF may represent an early phase or incomplete form of CTD, the clinical features and biomarkers of CTD must be regularly evaluated for all patients with IPAF. Multidisciplinary discussion for the early identification of IPAF followed by the implementation of rheumatologist–pulmonologist combined care is also recommended [21,22].

The standards for managing patients with IPAF have yet to be firmly established. The current treatment strategies for IPAF are mainly implemented on the basis of previous experience with CTD-associated ILD, for which corticosteroids and immunosuppressants are regarded as the mainstay of treatments [23]. No randomized controlled trials have been conducted to verify the efficacy of immunosuppressants for IPAF. A recent review [24] that examined case series and retrospective studies has suggested that mycophenolate mofetil and azathioprine can be used as first-line steroid-sparing agents, and rituximab, intravenous cyclophosphamide, and calcineurin inhibitors can be used for refractory diseases. Studies have verified that mycophenolate mofetil, rituximab, and cyclophosphamide are associated with improvements in FVC [25,26,27]. Antifibrotics have also been applied for patients with IPAF who exhibited a usual interstitial pneumonia (UIP) feature. Two randomized controlled trials investigated the use of antifibrotics (pirfenidone and nintedanib) targeting progressive fibrosing-ILD; the patients examined in these trials included a subset of patients who met the IPAF criteria, and both trials reported a slower decrease in FVC relative to placebos [28,29]. Similar results were found in the recent retrospective study showing pirfenidone might improve FVC in patients with IPAF, in addition to a lower prednisone dose after 12 months of treatment [30]. However, for our patient, maintenance therapy with azathioprine, hydroxychloroquine, and corticosteroids was ineffective in preventing lung function decline and acute exacerbation. Therefore, large prospective studies are required to ascertain the efficacy and safety of immunosuppressants and antifibrotics for patients with IPAF.

Our patient presented with respiratory failure that necessitated endotracheal intubation and mechanical ventilation at the initial diagnosis of IPAF, and she subsequently experienced recurrent episodes of acute exacerbation, which were all successfully ameliorated with methylprednisolone pulse therapy. A similar case with IPAF and respiratory failure was reported in a recent review [13]; the patient was successfully salvaged with methylprednisolone pulse therapy and six doses of cyclophosphamide. Based on the promising results of these two cases, glucocorticoid pulse therapy combined with cyclophosphamide may be an effective strategy for exacerbation of IPAF but warrants further studies.

The findings relating to the prognostic factors in investigated cohorts have been inconsistent. A systematic review and meta-analysis was conducted by Kamiya et al., who reviewed 12 retrospective cohorts with a total of 656 individuals; the researchers reported that only age was a prognostic factor for all-cause mortality in IPAF, with a hazard ratio of 1.06 (95% confidence interval of 1.04 to 1.07) [31]. Other potential unfavorable predictors included being male, smoking, having a UIP pattern, having lower FVC, and having lower DLCO; however, the quality of the published evidence is low. Despite the absence of UIP morphology, the characteristics of older age, decreased FVC, decreased DLCO, and anti-Ro52 may partially explain our patient’s rapid progression, recurrent exacerbations, and ultimate outcome of mortality. The detection of anti-Ro52 in myositis appears to be associated with a severe course and high frequency of rapidly progressive ILD [32,33]. The prognostic implication of anti-Ro52 in IPAF warrants further studies.

In spite of the limited data on the clinical outcome of IPAF, several studies have compared the exacerbation risk and survival rates of IPAF and idiopathic pulmonary fibrosis (IPF). In a retrospective study conducted by Lim et al., patients with IPAF had longer survival (mean survival time of 73.3 months vs. 52 months, *p* < 0.001). They also had fewer episodes of exacerbation (25.9% in a 10-year period vs. vs. 35.4% for patients with IPF, *p* < 0.001) [10]. In a prospective study conducted by Sebastiani et al., the estimated 5-year survival rate of patients with IPAF was significantly higher than that of patients with IPF (69.5% ± 7.8% vs. 36.8% ± 5.9%, *p* < 0.001) [20]. Furthermore, the 3-year transplant-free survival rate of MSAs-positive IPAF was analyzed in a retrospective study by Graham et al., who reported that the survival rate of the MSAs-positive IPAF cohort was similar to that of the idiopathic inflammatory myopathy-ILD cohort; they also suggested that MSAs should be removed from the IPAF criteria, and that MSAs-positive IPAF should be managed as an idiopathic inflammatory myopathy-ILD [34].

The association between idiopathic inflammatory myopathy and malignancy is well established. Cancer can be diagnosed before, concomitant with, or after the diagnosis of inflammatory myopathy. The relapse or development of myopathy is also reported to be associated with cancer relapse [35]. No study has investigated the relationship between malignancy and IPAF with myositis-associated antibodies. Our patient’s endometrial cancer was diagnosed 2 years before she developed IPAF, and another malignancy with left neck lymph node metastasis was detected 15 months after initial presentation. We contend that patients with IPAF who have myositis-associated antibodies should receive careful screening for malignancy at presentation in addition to long-term surveillance, particularly for patients with rapid progression or recurrent exacerbations.

## 4. Conclusions

We presented a patient with IPAF who had a rapid progressive course. Her exacerbations were successfully treated with methylprednisolone pulse therapy, but maintenance therapy failed to control her disease. The optimal treatments for exacerbation and stable disease in IPAF require further clarification. The patient eventually experienced evolution into polymyositis with discovery of underlying malignancy. A longitudinal follow-up for the clinical features and biomarkers of CTD is highly recommended for all patients with IPAF.

## Figures and Tables

**Table 1:** Evolution into connective tissue diseases in patients with IPAF.

Study	Patients (n)	Evolution into CTDs (n, %)	Numbers of Specific CTDs	Median Follow-Up Time	Reference
Ito et al. 2017	98	12 (12.2%)	7 Rheumatoid arthritis 2 Systemic sclerosis 1 Systemic lupus erythematosus 1 Sjögren syndrome and systemic sclerosis 1 Dermatomyositis and systemic sclerosis	4.5 years	[8]
Kim et al. 2020	109	13 (11.9%)	4 Rheumatoid arthritis 9 Not reported	3.8 years	[9]
Sambataro et al. 2020	32	6 (18.8%)	3 Polymyositis 1 Systemic sclerosis 1 Sjögren syndrome 1 Polymyositis and Sjögren syndrome	1 year	[16]
Alevizos et al. 2020	50	8 (16%)	3 Systemic sclerosis 2 Rheumatoid arthritis 2 ANCA-associated vasculitis 1 Polymyositis	5.2 years	[17]
Sebastiani et al. 2020	52	7 (13.5%)	4 Sjögren syndrome 2 Rheumatoid arthritis 1 Polymyositis	2.3 years	[18]
Decker et al. 2022	70	18 (26%)	9 Antisynthetase syndrome 8 Systemic sclerosis 1 Overlap myositis	6.4 years	[19]
Jiwrajka et al. 2022	60	6 (10%)	3 Antisynthetase syndrome 1 Rheumatoid arthritis 1 Systemic sclerosis 1 Sjögren syndrome	3 years	[20]

CTD = connective tissue disease; IPAF = interstitial pneumonia with autoimmune features; ANCA = antineutrophil cytoplasmic antibodies.

## Data Availability

Not applicable.
